# A multimodal dialog approach to mental state characterization in clinically depressed, anxious, and suicidal populations

**DOI:** 10.3389/fpsyg.2023.1135469

**Published:** 2023-09-11

**Authors:** Joshua Cohen, Vanessa Richter, Michael Neumann, David Black, Allie Haq, Jennifer Wright-Berryman, Vikram Ramanarayanan

**Affiliations:** ^1^Clarigent Health, Mason, OH, United States; ^2^Modality.AI, Inc., San Francisco, CA, United States; ^3^Department of Social Work, College of Allied Health Sciences, University of Cincinnati, Cincinnati, OH, United States; ^4^Otolaryngology - Head and Neck Surgery (OHNS), University of California, San Francisco, San Francisco, CA, United States

**Keywords:** machine learning, multimodal dialog systems, speech features, natural language processing, facial features, suicide, depression, anxiety

## Abstract

**Background:**

The rise of depression, anxiety, and suicide rates has led to increased demand for telemedicine-based mental health screening and remote patient monitoring (RPM) solutions to alleviate the burden on, and enhance the efficiency of, mental health practitioners. Multimodal dialog systems (MDS) that conduct on-demand, structured interviews offer a scalable and cost-effective solution to address this need.

**Objective:**

This study evaluates the feasibility of a cloud based MDS agent, Tina, for mental state characterization in participants with depression, anxiety, and suicide risk.

**Method:**

Sixty-eight participants were recruited through an online health registry and completed 73 sessions, with 15 (20.6%), 21 (28.8%), and 26 (35.6%) sessions screening positive for depression, anxiety, and suicide risk, respectively using conventional screening instruments. Participants then interacted with Tina as they completed a structured interview designed to elicit calibrated, open-ended responses regarding the participants' feelings and emotional state. Simultaneously, the platform streamed their speech and video recordings in real-time to a HIPAA-compliant cloud server, to compute speech, language, and facial movement-based biomarkers. After their sessions, participants completed user experience surveys. Machine learning models were developed using extracted features and evaluated with the area under the receiver operating characteristic curve (AUC).

**Results:**

For both depression and suicide risk, affected individuals tended to have a higher percent pause time, while those positive for anxiety showed reduced lip movement relative to healthy controls. In terms of single-modality classification models, speech features performed best for depression (AUC = 0.64; 95% CI = 0.51–0.78), facial features for anxiety (AUC = 0.57; 95% CI = 0.43–0.71), and text features for suicide risk (AUC = 0.65; 95% CI = 0.52–0.78). Best overall performance was achieved by decision fusion of all models in identifying suicide risk (AUC = 0.76; 95% CI = 0.65–0.87). Participants reported the experience comfortable and shared their feelings.

**Conclusion:**

MDS is a feasible, useful, effective, and interpretable solution for RPM in real-world clinical depression, anxiety, and suicidal populations. Facial information is more informative for anxiety classification, while speech and language are more discriminative of depression and suicidality markers. In general, combining speech, language, and facial information improved model performance on all classification tasks.

## 1. Introduction

Globally, ~301 million people and 280 million people were affected by anxiety and depression in 2019, respectively.^1^ According to the World Health Organization, over 700,000 people die by suicide every year, with more than 20 suicide attempts per suicide death (World Health Organization, 2021). In 2022 in the United States (US), 5.0% of adults report regular feelings of depression, and 12.5% report regular feelings of worry, nervousness, or anxiety.^2^ Regarding the frequency of suicidal thoughts in the US, 3.7% of adults had serious thoughts of suicide in 2021. Initial estimates of the impact of the COVID-19 pandemic show more than a 25% increase in mental disorders, worldwide (World Health Organization, 2022). As the prevalence of these conditions increases, technological solutions are needed to more efficiently identify, monitor, and manage these conditions.

The identification and monitoring of mental health conditions related to depression, anxiety, and suicide risk often rely on self report from individuals or evaluation from a trained professional. Self report scales, such as the Patient Health Questionnaire-9 Item (PHQ-9) for depression (Kroenke et al., 2001) or the Generalized Anxiety Disorder-7 Item (GAD-7) for anxiety (Spitzer et al., 2006), have reported excellent sensitivity and specificity but rely on the honesty of a patient and typically only screen for a single condition, requiring additional time to screen for more than one condition. For suicide risk, a 2017 meta analysis suggests current methods of predicting death by suicide are no better than random chance, and recommends other techniques such as machine learning (ML) to improve predictive capabilities (Franklin et al., 2017).

In clinical settings, information from a patient's visual appearance and body language, verbal communications, and speech may aid clinicians' diagnoses. More recently, these signals have been combined with supervised ML as biomarkers to identify the presence of mental health conditions (Ramanarayanan et al., 2022). In this context, signals from different modalities (e.g., speech or visual inputs) are transformed into features, leading to 100's to 1,000's of data points that describe aspects of the signal. For example, speech (S) features describe characteristics of an acoustic signal, such as pitch or intensity. Facial features (F) describe aspects of face movements, such as the number of eye blinks per second or the speed of the lower lip and jaw center. Text (T) features are derived from a patient's language and may capture relevant semantic information. During supervised ML, features are paired with a clinical label, such as having a condition (case) or not (control), and then used to train a model to allow the discovery of patterns from the data for classification.

Reviews of articles using ML with SFT features for the identification of depression, anxiety, and suicide risk indicate good to excellent model performance, with many investigators reporting areas under the receiver operating characteristic curve (AUC) in the range of 0.7–0.9 (Cummins et al., 2015, 2018; Arif et al., 2020; Bernert et al., 2020; Neumann et al., 2020; Kusuma et al., 2022). Comparatively, under realistic clinical conditions, many traditional mental health diagnostic checklists perform with AUCs in the range of 0.7–0.8 (Rice and Harris, 2005; Youngstrom, 2013). While ML models appear to perform with a similar discriminative ability as traditional methods, they face unique challenges. A key challenge is model overfitting, which occurs when a model learns from idiosyncrasies of a dataset as opposed to clinically meaningful variables. This leads to overly optimistic estimates of model performance and may result in a model that is overly sensitive to specific expressions of mental health conditions, reducing its effectiveness when symptoms are expressed differently (Berisha et al., 2022). Mitigation strategies include cross-validation, regularization, or using models with fewer parameters. Additionally, there are significant challenges to generalizability. Work by Botelho et al. (2022) shows a high degree of separability among six popular speech datasets, demonstrating the limitation that a model trained on one population or dataset might not accurately predict outcomes in another. This necessitates rigorous external validation and diverse, representative data collection. Biases in the dataset represent another source of error. If the training data predominantly represents a specific demographic or cultural group, the model may not perform as well on other groups, leading to misdiagnosis or underdiagnosis. Furthermore, Berisha et al. (2022) recently reported a negative association between model performance and sample size among 77 publications on speech-based ML for the identification of dementia, attributing this to not only model overfitting but also publication bias. This finding underscores the importance of transparency and balanced reporting in research publications. Taken together, these issues emphasize the importance of not solely focusing on classification performance but also on selecting clinically meaningful and generalizable features, ensuring a representative dataset, and employing robust validation methodologies. While ML shows promising potential in mental health diagnostics, these challenges must be recognized and addressed to maximize its clinical utility.

Previous research related to this work has found a semi-structured, in-person interview promising for the collection of SFT features to be used with ML models for the identification of suicide risk (Pestian et al., 2010, 2016, 2017; Laksana et al., 2017; Cohen et al., 2020, 2022; Wright-Berryman et al., 2023). In these studies, trained staff (therapists, clinical research coordinators, or licensed behavioral health clinicians) recorded a semi-structured interview with hundreds of suicidal or non-suicidal participants in emergency departments, psychiatric units, and in-school therapy settings with adolescents and adults. Support vector machine (SVM) models were trained to identify suicidal vs non-suicidal participants, with AUCs ranging from 0.69 to 0.93 depending on the features and cross-validation approach used (Pestian et al., 2016, 2017). Notably, two of these investigations included an external validation of the models developed with separately collected corpora (Cohen et al., 2020, 2022). It is also important to note that while these studies involved hundreds of participants, there is no universally accepted minimum sample size for ML analyses. The required sample size can vary greatly depending on the complexity of the model, the number of features, the variability in the data, and the specific research question being addressed. Some studies have successfully applied ML techniques with as few as 60 sessions (Pestian et al., 2016).

While these initial results are encouraging for the use of SFT features for the identification of suicide risk in clinical settings, the procedures relied on trained staff to conduct the interview. There is a shortage of mental health professionals (Satiani et al., 2018), which may limit the uptake of technology requiring more of their time. Therefore, techniques to accurately and autonomously screen for mental health concerns are needed. One option may be to use multimodal dialog systems (MDS), which have recently been developed for remote health screening and monitoring. For example, DeVault et al. (2014) presented the SimSensei Kiosk, a virtual human interviewer specifically built to render clinical decision support. It captures verbal and non-verbal features to extract distress indicators correlated with mental conditions such as depression. Lisetti et al. (2015) presented results of a large-scale effort building a virtual health assistant for “brief motivational interventions,” for example, interviews about a subject's drinking behavior. The described system uses text input from the subject's keyboard (or, alternatively, a speech recognition hypothesis) along with facial expression features to determine next steps in the interaction. In addition to cost reduction and scalability, MDSs may reduce participants' fear associated with the perception of being judged (Cummins et al., 2015). Gratch et al. (2014) found that participants felt more comfortable disclosing personal information with an agent that was framed as autonomous as opposed to one that was framed as human-controlled.

For the present study, the Modality service, a cloud-based MDS (Suendermann-Oeft et al., 2019; Ramanarayanan et al., 2020) was used to conduct automated, structured interviews with participants. Neumann et al. (2020) recently demonstrated the utility of the Modality MDS in differentiating people with mild, moderate and severe depression, and similar studies have also been conducted in ALS (Neumann et al., 2021), Parkinson's disease (Kothare et al., 2022), schizophrenia (Richter et al., 2022), and autism (Kothare et al., 2021). The Modality MDS can be used with widely available endpoints such as smartphones and laptops as opposed to the dedicated, locally administered hardware used in other studies. Speech, facial, and language data was collected by the MDS for feature analysis and ML classification of depression, anxiety, and suicide risk. Overall, we found participants accepting of the technology and procedures, and ML models using a combination of features led to the greatest discriminative ability.

This case-control study sought to (1) examine the feasibility of collecting a mental health interview with an MDS with participants with and without depression, anxiety, and suicide risk, (2) evaluate candidate features for the identification of these conditions, and (3) internally validate models trained to identify each condition with different modalities (speech, facial, and text).

## 2. Methods

### 2.1. Data

Sixty-eight participants enrolled in the study between October 2021 and April 2022, providing a total of 73 sessions. Notably, participants were allowed (but not required) to participate again after 2 weeks, out of whom five participants chose to take part in another session each. The PHQ-9 to measure depression (Kroenke et al., 2001), the GAD-7 to measure anxiety (Spitzer et al., 2006), and the Columbia-Suicide Severity Rating Scale (C-SSRS) Screener (Posner et al., 2011) to measure suicide risk were collected in all sessions. Participant demographics and distributions of case sessions are shown in Table 1. For a more complete picture of the study participants, statistics of control participants and additional demographic information are available in Supplementary Table 1.

**Table 1:** Participant descriptive statistics and case session summaries.

			**Case sessions**		
**Variable**	**Participants**	**Sessions**	**PHQ-9** ≥10	**GAD-7** ≥10	**C-SSRS** ≥**Mod**.
Count (%)	68 (100.0%)	73 (100.0%)	15 (20.6%)	21 (28.8%)	26 (35.6%)
Average age (SD)	38.8 (14.7)	38.7 (14.7)	39.3 (13.3)	34.5 (13.1)	38.8 (15.7)
Average interview length (min) (SD)	9.6 (2.2)	9.3 (2.3)	9.7 (2.5)	9.0 (2.4)	9.7 (2.2)
Average word count (SD)	917.0 (302.06)	925.0 (309.9)	912.1 (374.2)	899.1 (308.5)	964.1 (316.2)
**Sex**					
Male (%)	15 (22.1%)	16 (21.9%)	3 (4.11%)	6 (8.2%)	9 (12.3%)
Female (%)	52 (76.5%)	56 (76.7%)	12 (16.4%)	15 (20.6%)	16 (21.9%)
Prefer not to answer	1 (1.5%)	1 (1.4%)	- (-%)	- (-%)	1 (1.4%)
**Race**					
White or Caucasian (%)	50 (73.5%)	54 (74%)	12 (16.4%)	17 (23.3%)	21 (28.8%)
Black or African American (%)	10 (14.7%)	11 (15.1%)	2 (2.7%)	3 (4.1%)	2 (2.7%)
Asian (%)	5 (7.4%)	5 (6.9%)	- (-%)	1 (1.4%)	- (-%)
Other (%)	3 (4.4%)	3 (4.1%)	1 (1.4%)	- (-%)	3 (4.1%)

Criteria for participant recruitment were: (1) age ≥ 18, (2) able to provide informed consent, (3) English as a primary language, and (4) located in the United States. Recruitment for the study was done via ResearchMatch, a national health volunteer registry that was created by several academic institutions and supported by the U.S. National Institutes of Health as part of the Clinical Translational Science Award program. ResearchMatch has a large population of volunteers who have consented to be contacted by researchers about health studies for which they may be eligible. For this study, we specifically targeted individuals who had self-selected to be contacted by studies related to depression, anxiety, and suicide risk. This targeted recruitment strategy was designed to ensure a sufficient number of participants with the conditions of interest. Review and approval for this study and all procedures was obtained from our commercial Institutional Review Board. All participants gave informed consent in accordance with the Declaration of Helsinki before they participated in the study. Participants received a $15 gift card for each session they completed.

#### 2.1.1. Study staff

The study staff was composed of three clinical research coordinators (CRC) who are all mental health practitioners or graduate-level students in the mental health field. They are extensively trained in all study procedures, human subjects protection, good clinical practice, and crisis management procedures. The CRCs oversaw all study procedures.

### 2.2. Study design

**Figure 1:**
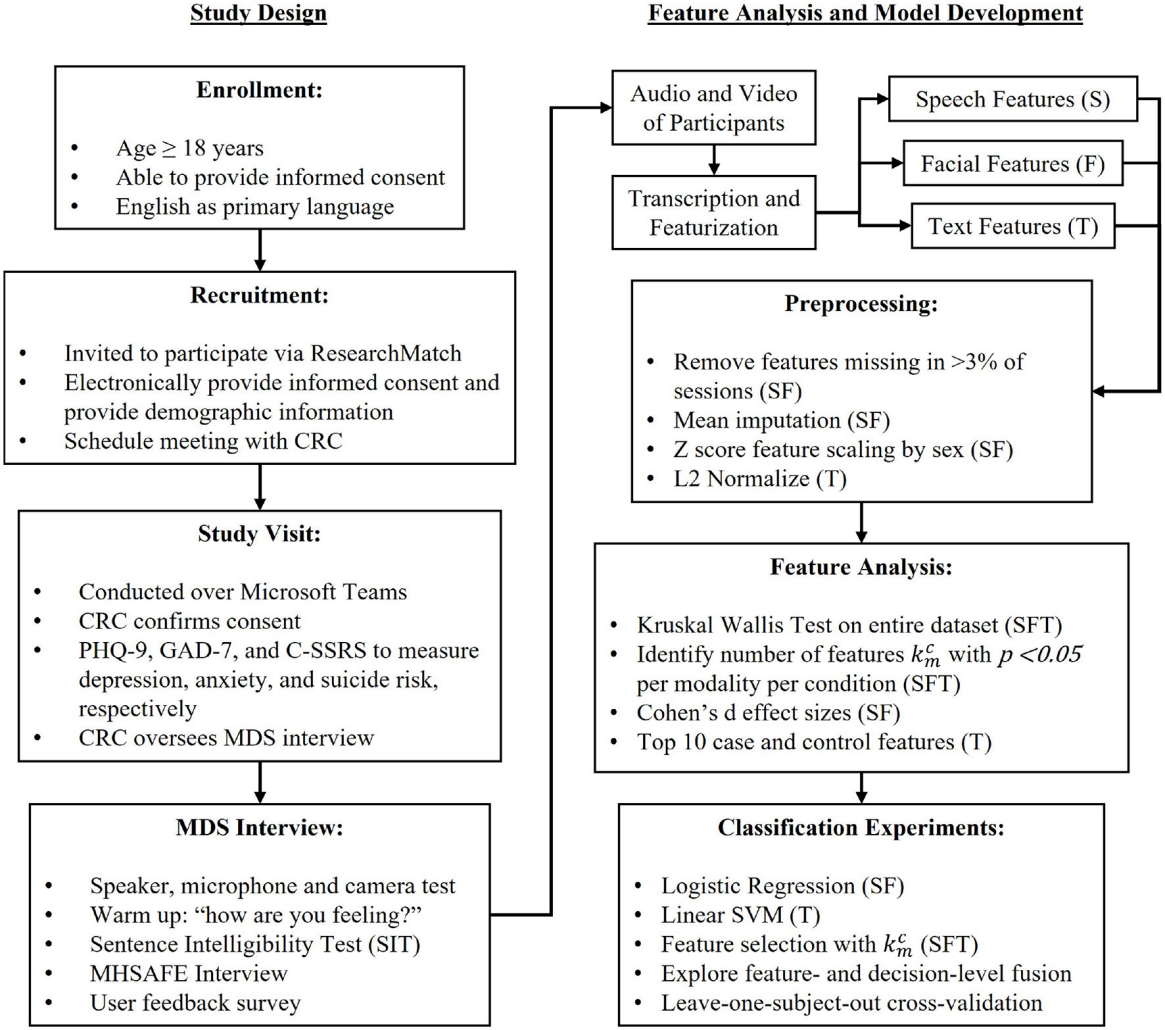
Schematic of study and modeling procedures.

Participants invited through ResearchMatch completed informed consent and demographic information electronically and scheduled a remote study session time with a CRC to meet via the video conferencing platform Microsoft Teams. During the study session, the CRC confirmed participant consent and that they understood the study procedures and their rights as participants, and then administered the PHQ-9, GAD-7, and the C-SSRS Screener. These instruments have been administered via video conferencing platforms in a variety of studies. Figure 1 outlines the study procedures.

The PHQ-9 is a rigorously tested, reliable and valid instrument for depression in adults, with a sensitivity and specificity of 88%, corresponding with a threshold score ≥10 out of 27, which includes “Moderate,” “Moderately Severe,” and “Severe” levels of depression (Kroenke et al., 2001). Similarly, the GAD-7 has been widely tested with adults to measure anxiety, with a sensitivity and specificity of 89 and 82%, respectively, corresponding with a threshold score ≥10 out of 21, which includes “Moderate” and “Severe” levels of anxiety (Spitzer et al., 2006). The C-SSRS Screener is a structured interview which has demonstrated high sensitivity and specificity for classifying suicidal ideation and behaviors in a multi-site emergency department study (Posner et al., 2011). The screener asks six questions about the past month to measure suicidal ideation and suicidal behaviors on an ordinal scale, with a final question about lifetime suicidal behavior (more than 3 months ago). The C-SSRS Screener designates suicide risk as “None” if all questions are answered negatively, “Low” if passive suicidal ideation is present, “Moderate” if suicidal ideation with a method OR lifetime suicidal behavior is present, and “High” if suicidal ideation with intent (with or without a method) OR suicidal behavior in the past 3 months is present. In this study a severity threshold ≥ “Moderate” was used for the binary identification of all conditions to maximize sensitivity and specificity of the instruments. Table 2 is a summary of the assessments and scores used for case definitions.

**Table 2:** Summary of completed assessments, associated mental state measured, and case definition for model development.

**Assessment**	**Mental state**	**Case definition**
PHQ-9	Depression	Total ≥10
GAD-7	Anxiety	Total ≥10
C-SSRS screener	Suicidal risk	Risk ≥ Moderate

For participant safety, all participants received wellness resources such as the 988 Suicide and Crisis Lifeline and the Crisis Text Line. The 988 National Suicide Prevention Lifeline and the Crisis Text Line are U.S.-based single line immediate access to trained crisis counselors. For participants that score “High” risk on the C-SSRS Screener, a more comprehensive contingency plan was followed, including asking additional questions about their mental state, access to lethal means, engagement in mental health services, and protective factors. In the event of imminent risk, the contingency safety plan included a warm hand-off to the 988 Suicide and Crisis Lifeline and/or a call to 911. No participants in this study were at imminent risk and required following of the contingency safety plan.

Following the PHQ-9, GAD-7, and C-SSRS, the CRCs provided a link to the MDS, a web-based program that accesses the participant's computer's microphone and webcam to record their voice and facial video. To supervise this section of the study, CRCs instructed participants to share their computer's screen and audio. The CRC then muted their microphone and turned off their webcam.

Before participants start their conversation with the virtual agent, Tina—implemented via a scalable, cloud-based MDS to conduct automated structured interactions (Suendermann-Oeft et al., 2019; Ramanarayanan et al., 2020)—tests of the speaker, microphone, and camera need to be passed to ensure that the participants' devices are correctly configured so that the collected data has sufficient quality. Once all device tests pass, Tina guides participants through an interactive interview.

In each participant's first session, Tina introduced the graphical interface and asked the participant to read a sentence, taken from a speech intelligibility test (SIT) corpus. At the start of every interview, Tina first asked participants “how they are feeling today” as a warm up question. Participants then began a semi-structured inteview (renamed MHSAFE—hope, secrets, anger, fear, and emotional pain–from the “Ubiquitous Questionnaire”). The MHSAFE interview has been used in previous studies with human interviewers to collect language for ML models to identify suicide risk (Pestian et al., 2010, 2016, 2017; Laksana et al., 2017; Cohen et al., 2020, 2022). The interview asks participants open-ended questions about five topics: hope, secrets, anger, fear, and emotional pain (Pestian, 2010; Cohen et al., 2020, 2022). In the present study, for each topic, Tina asks if they have that topic and how that makes them feel, for example, “do you have hope and how does that feel?” The question about secrets is not intended for participants to reveal what their secrets are, but to gather information about whether they are keeping secrets at all, and how they feel about this. The SIT task and warm up question from the beginning were included in the analysis, because these speech samples may contain useful features in addition to the MHSAFE interview.

Tina is equipped with a voice activity detection system to measure the length of participant responses. To collect enough language for analysis, Tina required a minimum of 1 min of speech for each topic of the MHSAFE interview. Participants that did not speak for the minimum amount of time were nudged up to two times to tell Tina more about that topic. Tina moved onto to the next question if after two nudges the participant's speaking time for that questions was still <1 min. The recorded audio files were manually transcribed using a HIPAA-compliant service.

### 2.3. User feedback

For user feedback, we used two forms of data collection, a qualitative questionnaire (likes/advantages, dislikes/disadvantages, and improvements) and a five-question survey with Likert scale responses, shown in Table 3. Qualitative data were analyzed using thematic analysis. Two investigators coded the responses and annotated the emerging themes. Likert scale responses were analyzed using frequency distribution, mean, and standard deviation. Student's *t*-tests were performed with SciPy's *ttest_ind* function to identify any statistically significant differences between case and control groups for Likert scale responses.

**Table 3:** Post interview survey.

**Item**	**Survey questions**
**Likert scale questions: 1 = most negative to 5 = most positive**	
1.	How did it feel to express your emotions of your hope, secrets, anger, fear, and emotional pain to a virtual assistant?
2.	How honest were you in your responses to the virtual assistant?
3.	How comfortable were in your responses to the virtual assistant?
4.	What was your impression of the virtual assistant in terms of visual appearance and voice?
5.	What was your impression of the virtual assistant in terms of pace of interview including interruptions and pauses from the virtual assistant, and your time to respond?
**Open-ended questions:**	
6.	What did you like about Tina?
7.	What did you not like about Tina?
8.	What could be improved with this experience?

### 2.4. Data preprocessing and featurization

All analysis was performed using the Python programming language (version 3.9.12; Van Rossum and Drake, 1995). The following open-source Python libraries were also used: Pandas (version 1.4.2; McKinney, 2010; The Pandas Development Team, 2020), Numpy (version 1.22.3; Oliphant, 2007; Van Der Walt et al., 2011), scikit-learn (version 1.0.2; Pedregosa et al., 2011), Matplotlib (version 3.5.1; Hunter, 2007), and SciPy (version 1.8.0; Virtanen et al., 2020). For calculating effect sizes, we also used the R package effsize (version: 0.7.6; Torchiano, 2020) and the rpy2 interface (version 2.9.4).^3^

In our methodology, three modalities—acoustic (speech), facial, and textual—were examined, each contributing a distinct set of features to our models and are described in more detail below.

#### 2.4.1. Speech features

For the acoustic speech analysis, a variety of commonly established measures for clinical voice analysis were extracted (France et al., 2000; Mundt et al., 2007, 2012). These include *timing measures*, such as percentage of pause time (PPT), *frequency domain measures*, such as fundamental frequency (F0) and jitter, *energy-related measures*, such as intensity and shimmer as well as the harmonics-to-noise ratio (HNR) as a measure for *voice quality*. All measures were extracted with Praat (Boersma and Van Heuven, 2001). Table 4 lists all features. More detailed descriptions of speech features are available in Supplementary Table 2.

**Table 4:** Overview of speech, facial, and text features.

	**Domain**	**Features**
Speech	Energy	Shimmer (%), signal-to-noise ratio (dB)
	Timing	Speaking and articulation duration (sec.), percent pause time (PPT, %)
	Voice quality	Harmonics-to-noise ratio (HNR, dB)
	Frequency	Mean, max., min. fundamental frequency F0 (Hz), jitter (%)
Facial	Mouth (distances)	Lip aperture/opening, lip width, mouth surface area, Mean symmetry ratio between left and right half of the mouth
	Movement	Velocity, acceleration, jerk, and speed of lower lip and jaw center
	Eyes	Number of eye blinks per sec., eye opening, vertical displacement of eyebrows
Text	TF-IDF	$\frac{\text{Count of n-gram in interview}}{\text{Count of interviews containing n-gram}}$

For facial features, functionals (minimum, maximum, and average) are applied to produce one value across all video frames of an utterance.

#### 2.4.2. Facial features

**Figure 2:**
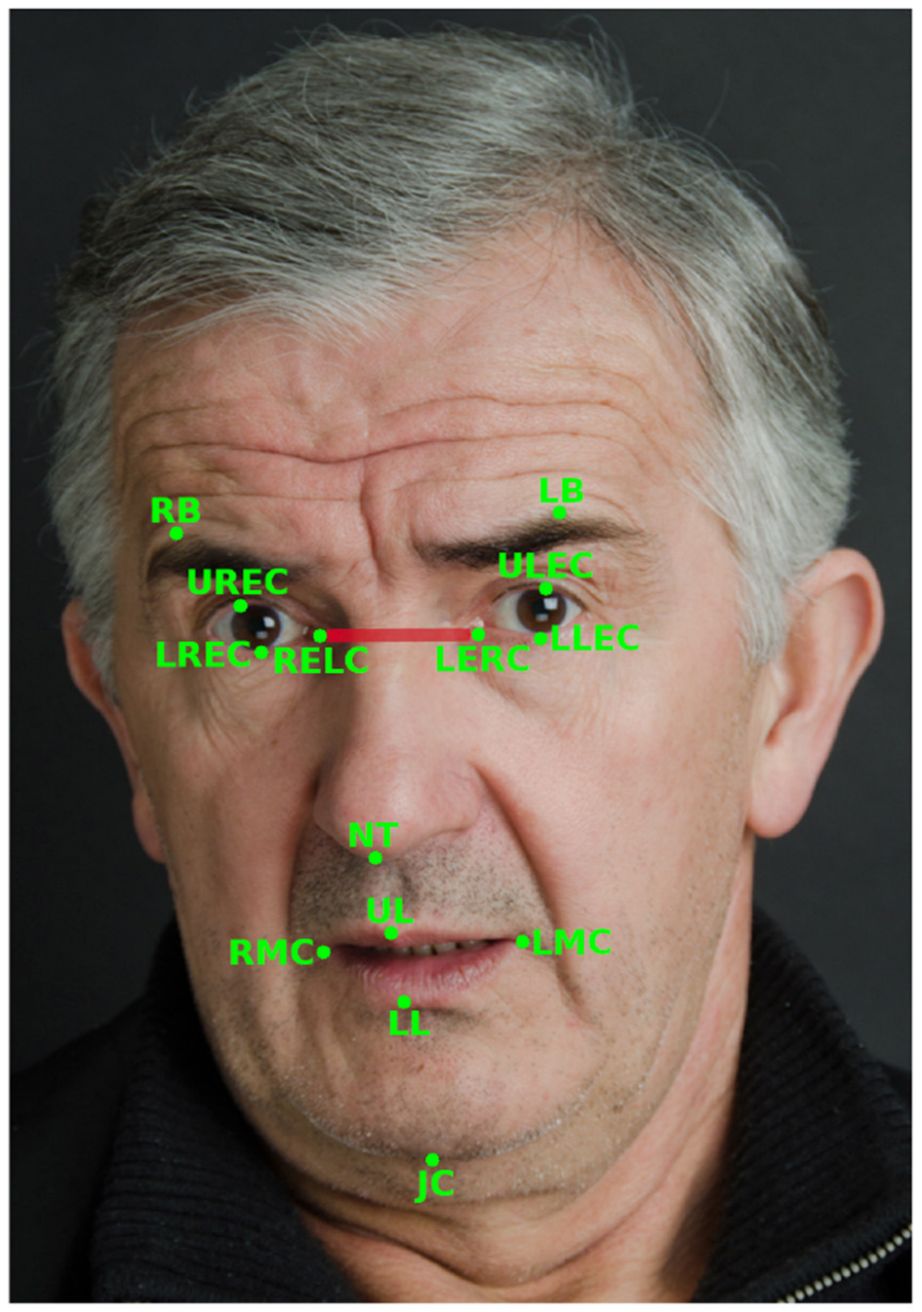
Illustration of the 14 facial landmarks used to calculate the facial features used in this study and the inter-caruncular distance (shown in red) between the inner canthi of the eyes (RELC and LERC). Adapted with permission from iStock Photo/meshaphoto.

The set of facial features is based on facial landmarks generated in real time by the MediaPipe Face Mesh algorithm (Kartynnik et al., 2019). For each user turn, the following algorithm is applied to compute features. First, MediaPipe Face Detection, which is based on BlazeFace (Bazarevsky et al., 2019), is used to determine the (x, y)-coordinates of the face for every frame. Then, facial landmarks are extracted using MediaPipe Face Mesh. We use 14 key landmarks to compute features like the speed and acceleration of articulators (jaw and lower lip), surface area of the mouth, and eyebrow raises (see Table 4). The key facial landmarks are illustrated in Figure 2. Lastly, the features are normalized by dividing them by the inter-caruncular or inter-canthal distance, which is the distance between the inner canthi of the eyes (see Figure 2 for a visual illustration), to handle variability across participant sessions due to position and movement relative to the camera (Roesler et al., 2022). More detailed descriptions of facial features are available in Supplementary Table 3.

#### 2.4.3. Text features

The natural language processing (NLP)/ML pipeline used in this study focused on the term frequency-inverse document frequency (TF-IDF) of unigrams (single words), calculated using scikit-learn's *TfidfVectorizer*. TF-IDF is a numerical statistic that reflects how often a term appears in a document (i.e., interview), while also taking into account how common the term is in the entire corpus of documents. This weighting scheme assigns higher importance to terms that are more distinctive to a particular document, and lower importance to terms that are common across many documents (Rajaraman and Ullman, 2011).

The text was preprocessed so all characters were lowercase and to remove any punctuation and non-letter characters. Language was tokenized by splitting on white spaces. Following the preprocessing steps, each session was subject to L2 normalization, a process designed to control for varying response lengths. L2 normalization, also known as Euclidean normalization, works by adjusting the values in the data vector so that the sum of the squares of these values equals one. Specifically, each value in the vector is divided by the Euclidean length (L2 norm) of the vector itself—the square root of the sum of the squared vector values.

#### 2.4.4. Missing data

Features may be missing if a participant skipped a segment or a technical issue arose. To handle missing speech and facial features, scikit-learn's *SimpleImputer* was used to replace the missing feature with its mean value for each cross-validation fold. Any feature missing from >3% of sessions was removed prior to model evaluation to ensure the robustness of our analyses and to avoid potential biases or inaccuracies that could arise from imputing a large amount of missing data.^4^

### 2.5. Feature analysis and classification experiments

Due to the limited size of our dataset, we performed feature analysis on the entire dataset to identify the *number* of significant features (but importantly, not which features). In other words, during classification experiments, we only specified the number of features, and not the specific features, per cross-validation fold to avoid information leakage across training and validation folds. To test statistical meaningfulness of the features, non-parametric Kruskal-Wallis (KW) tests (McKight and Najab, 2010) were conducted on the entire dataset for each feature, which test the hypothesis that feature medians are significantly different between cohorts (cases and controls) at the α = 0.05 level. In order to give equal weight to the features of individual participants, we selected only one session per user for this test. This results in $k_{m}^{c}$ number of features per modality (*m*), per condition (*c*). For speech and facial features, effect sizes were then calculated with Cohen's *d* (Cohen, 1988), which analyzes the direction and magnitude of effects between cohorts. Cohen's *d* was introduced to measure effect sizes in units of variability by dividing the difference of cohorts' means by the pooled standard deviation. Because TF-IDF featurization of participant language results in sparse matrices, we did not measure effect sizes, but instead extracted the top 10 case and control features by feature weight per condition from a linear SVM fit to the entire dataset after feature selection for the $k_{m}^{c}$ features determined by the KW test. Figure 1 includes a schematic of feature analysis and model development procedures.

Discrimination power was assessed by evaluating the classification performance using a logistic regression (LR) classifier for speech and facial features and a linear SVM for text features. These classifiers were selected for their relative simplicity and promising performance in previous studies (Pestian et al., 2016, 2017; Laksana et al., 2017; Cohen et al., 2020, 2022). To prune our high-dimensional feature set, the number of speech and facial input features for the classifier was determined by $k_{m}^{c}$. We selected the top $k_{m}^{c}$ features that resulted from a KW test on *n*−1 participants' session(s) in each classification fold. To ensure robustness and reliability of our results, we only reported ML experiments if they were based on at least five significant features. This threshold was set to avoid over-reliance on a small number of features or outliers, and to provide a more robust basis for classification.

The acoustic characteristics of male and female voices have been studied in detail and found to differ in a variety of variables such as pitch, voice quality, and timing measures (see, for example, Titze, 1989; Mendoza et al., 1996; Simpson, 2009). Furthermore, facial behavior as well as classification accuracy based on facial features was found to differ by gender (Dimberg and Lundquist, 1990; Drimalla et al., 2020). To ensure that analyses between case and control cohorts are unbiased with respect to widely reported differences between males and females, we standardized scores for speech and facial features by *z*-scoring for both groups separately.^5^

Both feature- and decision-level fusion were examined to identify any potential predictive benefits of including information from multiple modalities. During feature fusion, features are independently preprocessed and selected, and then merged into a single matrix prior to model development and evaluation. An LR classifier was used for feature fusion classification. Decision fusion involves independently training models on each modality or a combination thereof (e.g., speech and facial features combined together), and then combining outputs from each model through different rules. For decision fusion, LR classifiers were used for speech and facial features, while a linear SVM was used with text features. Model output combination rules considered include the minimum, maximum, and mean of all model output scores.

Models were trained using different feature combinations paired with each session's label as a case or control. Models were evaluated using a leave-one-subject-out cross-validation approach, where a model is iteratively trained on all but one participants' session(s). The features from the held out subject's session(s) were fed into the model and a probability for belonging to the case group was returned. When done iteratively, this results in a list of probabilities for each session to be compared to the true label to compute overall model performance metrics. Model performance was primarily evaluated with the AUC and Brier score. AUC values range from 0.5 (random chance) to 1.0 (perfect model). The Brier score is a measure of model calibration and ranges from 0 to 1 where low scores indicate less discrepancy between labels and predicted probabilities.

The selection of features in the cross-validation folds in the classification experiments based on a KW test on *n*−1 participants may differ from the result of the KW test on the entire cohort. To identify the most important features for each mental state in terms of robustness and generalizability across experiments and thus independence from participant partitions, we assessed these by determining the intersection of features that (a) were consistently selected across *all* cross-validation folds and (b) were found to be statistically significant in the KW test for the entire cohort. We then examined these features in more detail by reviewing previous research and by testing their association with the respective mental states. For the latter, Pearson correlations were calculated between the assessment total scores and the speech and facial features. A threshold of |*r*|≥0.2 and *p* < 0.05 was used to identify weak, but statistically significant correlations. We acknowledge that an |*r*| value of 0.2 is often considered a “small” effect size. However, in the context of our exploratory analysis with a relatively smaller dataset, we chose this threshold to highlight any potential weak, but statistically significant relationships that may warrant further investigation in larger studies. This approach allows us to focus on potentially clinically meaningful features and gives a more nuanced understanding of the data, rather than focusing exclusively on model performance.

## 3. Results

### 3.1. Feature analysis and classification experiments

**Figure 3:**
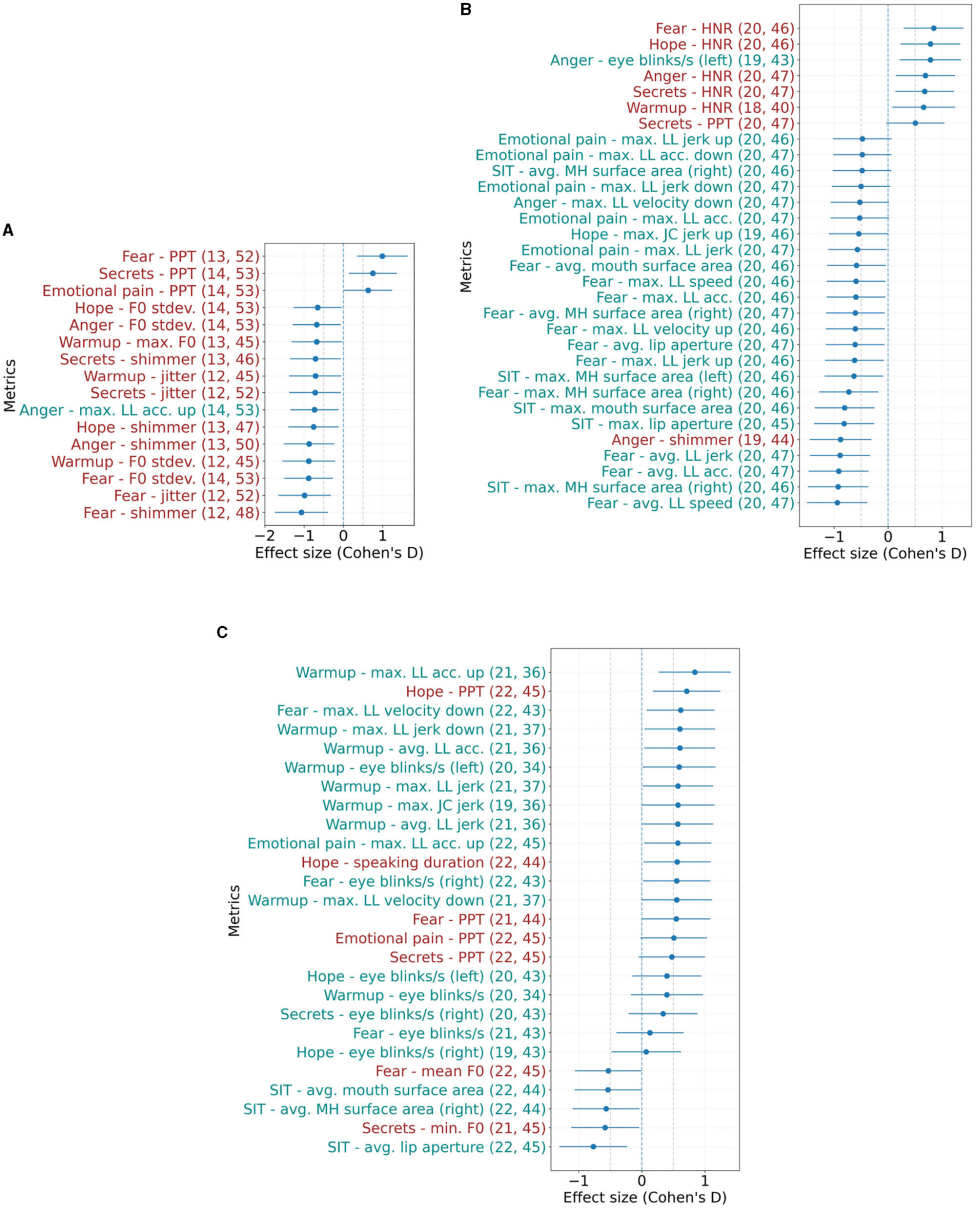
Effect sizes (Cohen's *d*) of speech and facial metrics that show statistically significant differences between controls and cases based on **(A)** PHQ-9 ≥10, **(B)** GAD-7 ≥10, and **(C)** C-SSRS (suicide risk) ≥*Moderate* at α = 0.05. Error bars show the 95% confidence interval. Positive values indicate features where cases had higher mean values than controls. Numbers in parentheses indicate the number of included samples for cases and controls. The respective task/ interview question is specified in the prefix. LL, lower lip; JC, jaw center; MH, mouth half; acc, acceleration. **(A)** Effect sizes based on PHQ-9. **(B)** Effect sizes based on GAD-7. **(C)** Effect sizes based on C-SSRS.

Figure 3 shows the effect sizes of the speech and facial features that are statistically significantly different between the respective cohorts. For PHQ-9 assessments, we find one facial and 15 speech features, as can be seen in Figure 3A. These features include a higher percent pause time as well as lower shimmer, jitter and F0 standard deviation for cases than controls. Conversely, for comparisons based on GAD-7 scores, more facial features (24) are evident than speech features (seven), as shown in Figure 3B. Similar to the GAD-7 assessments, seven speech and 24 facial features were found to be significant in the statistical analysis based on the C-SSRS scores, which is shown in Figure 3C. For each of the conditions examined in our study - depression, anxiety, and suicide—the top 10 text features (words) for both cases and controls were extracted using linear SVM models fit to the entire corpus, after the feature selection process. Importantly, these textual features do not overlap with or include acoustic or facial features - they are entirely separate. Table 5 provides a list of these top textual features for each condition.

**Table 5:** Top 10 text model features with the associated mental state.

**Mental state**	**Control features**	**Case features**
Depression	Friends, little, certain, think, right, this, some, unable, suffer, across	Homeless, nice, being, paper, very, NAME, times, following, poetry, should
Anxiety	Right, year, well, family, theres, friends, best, depression, far, thought	Lot, anxious, heart, his, mad, parents, everyone, another, sensitive, worst
Suicide	School, family, money, having, cry, these, changes, loss, worry, point	Yeah, very, at, fear, her, one, whether, still, when, seem

**Figure 4:**
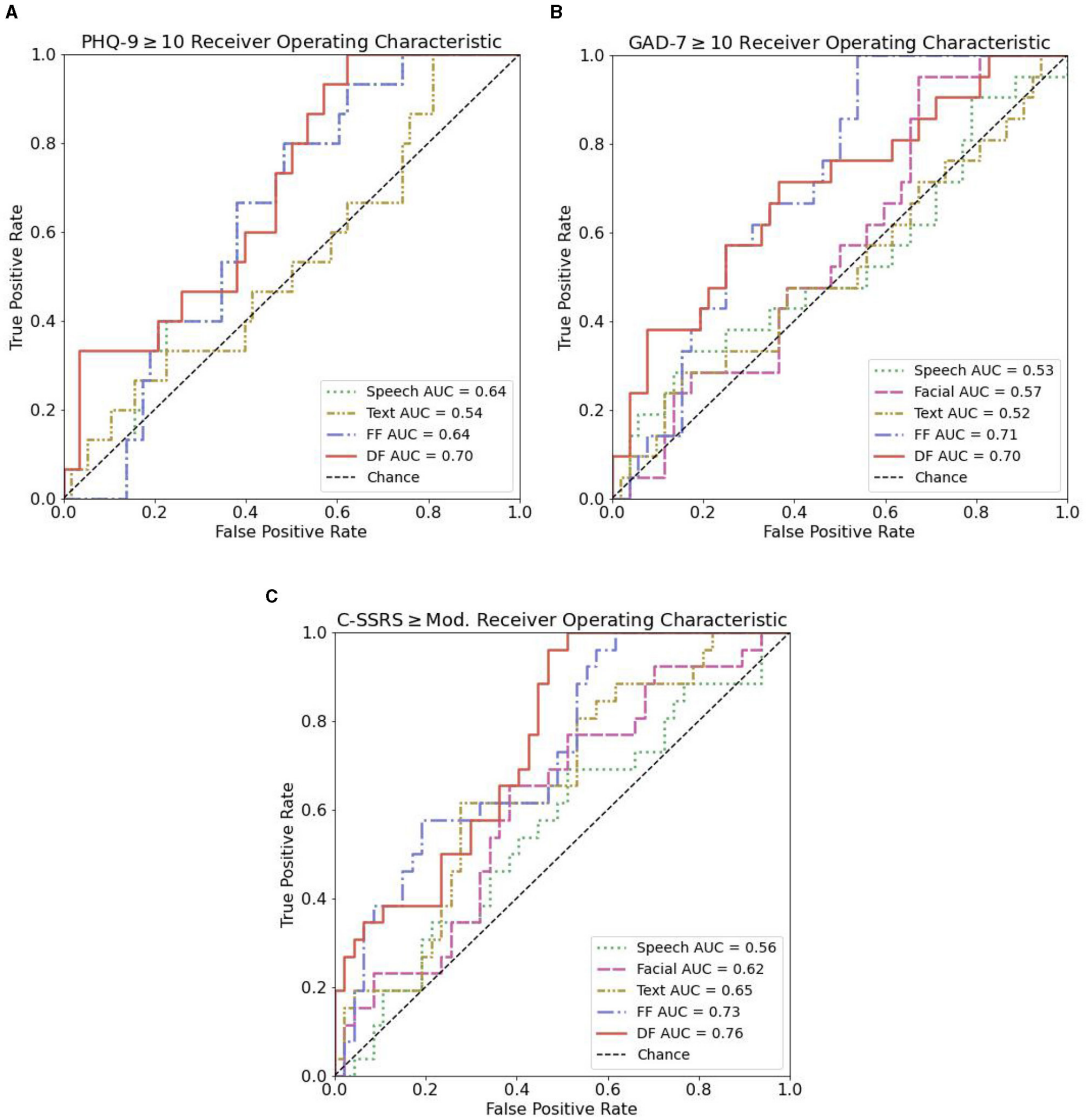
ROC curves for text, speech, facial, and the combination of speech and facial features in distinguishing controls from case participants. **(A)** ROC curves for PHQ-9 ≥10. **(B)** ROC curves for GAD-7 ≥10. **(C)** ROC curves for C-SSRS ≥ Mod.

Receiver operating characteristic (ROC) curves can be seen in Figure 4 for the identification of depression, anxiety, and suicide risk for single modalities, feature fusion, and decision fusion models. The results in terms of AUC and Brier score are shown in Table 6. Of the single-modality models, the best performance for depression occurred with speech features (AUC = 0.64; 95% CI = 0.51–0.78); for anxiety, facial features performed best (AUC = 0.57; 95% CI = 0.43–0.71); and for suicide risk, text features performed best (AUC = 0.65; 95% CI = 0.52–0.78). While these AUC values indicate that the models have some predictive power, it's important to highlight that an AUC of 0.5 would be equivalent to random chance and values in the range of 0.7–0.8 are often considered indicative of a good performing model. Thus, it can be seen that some of our single-modality models are performing at near-chance or sub-optimal levels (see Figure 4).

**Table 6:** Evaluation metric scores for all conditions for best performing models.

**Condition**	**Features**	**No. of features**	**AUC (95% CI)**	**Brier score**
Depression	Speech	15	0.64 (0.51–0.78)	0.22
		Text	450	0.54 (0.37–0.70)	0.26
Anxiety	Speech	7	0.53 (0.37–0.69)	0.23
		Facial	24	0.57 (0.43–0.71)	0.23
		Text	80	0.52 (0.36–0.67)	0.30
Su. Risk	Speech	7	0.56 (0.42–0.70)	0.26
		Facial	24	0.62 (0.49–0.76)	0.25
		Text	54	0.65 (0.52–0.78)	0.27
**Feature fusion (best performing combination)**				
Depression	Speech+Text	15+450	0.64 (0.51–0.78)	0.22
Anxiety	All	7+54+80	0.71 (0.59–0.83)	0.22
Su. Risk	All	7+24+54	0.73 (0.61–0.85)	0.22
**Decision fusion (best performing combination, min. scores)**				
Depression	Speech+Text	15+450	0.70 (0.56–0.84)	0.16
Anxiety	All	7+24+80	0.70 (0.56–0.83)	0.20
Su. Risk	All	7+24+54	0.76 (0.65–0.87)	0.21

Note that we do not report AUC for the depression classification task with facial features because only one feature remained after feature selection, with the resulting AUC less than chance, suggesting that facial features are not as useful as other modalities for depression discrimination in this study cohort.

In general, we found a combination of features or models improved discriminative ability, with a decision fusion of all models leading to the best overall performance in the identification of suicide risk (AUC = 0.76; 95% CI = 0.65–0.87). The best discriminative ability for depression (AUC = 0.70; 95% CI = 0.56–0.84) resulted from a decision level fusion of speech and text features. For anxiety, a feature-level fusion of all features performed best (AUC = 0.71; 95% CI = 0.59–0.83). The best performance for all decision-level fusion models resulted by selecting the minimum score of considered mode.

Further, we found the models' performance sensitive to the number of features fed into the classifier. Therefore, we performed a follow-up analysis to determine the optimal number of features for classification performance based on the AUC. We found that a small set of speech features with only three features is most beneficial for classifying depression, and similarly, a small set of nine features from combined speech and facial modalities is useful for classifying anxiety. The performance increased to a maximum AUC of 0.8 and 0.79, respectively.

Speech and facial features selected across experiments (KW tests on the entire sample and selected features in each leave-one-speaker-out cross-validation fold) are shown in Table 7. As can be seen for depression, speech frequency and timing metrics were found to be discriminative across experiments. Percent pause time in speaking about fear as well as about secrets is significantly higher in cases than in controls, while the standard deviation of F0 is lower. These results are in agreement with the conducted Pearson correlation analysis that revealed a statistically significant positive correlation (*p* = 0.025, *r* = 0.268) between percent pause time (fear) and PHQ-9 total scores as well as a negative correlation (*p*: 0.003, *r*: –0.346) between standard deviation of F0 (fear) and PHQ-9 total scores.

**Table 7:** Intersection of speech and facial features identified as statistically significant between respective cohorts for the entire sample and selected in every leave-one-speaker-out cross-validation fold.

**Mental state**	**Features**	**Effect sizes**	**Categories**
Depression	PPT (fear, secrets)	0.99, 0.75	Speech, timing
	F0 stdev. (fear)	−0.89	Speech, frequency
Anxiety	HNR (fear, hope, and secrets)	0.84, 0.78, 0.68	Speech, voice quality
	Max. jerk lower lip down (emotional pain)	0.5	Facial, movement
	Average speed lower lip (fear)	−0.94	Facial, movement
	Max. half mouth surface area right (SIT)	−0.93	Facial, mouth
	Average acc. lower lip (fear)	−0.92	Facial, movement
	Avg. jerk lower lip (fear)	−0.89	Facial, movement
	Max. lip aperture (SIT)	−0.81	Facial, mouth
	Max. mouth surface area (SIT)	−0.81	Facial, mouth
	Max. jerk lower lip up (fear, emotional pain)	−0.62, −0.48	Facial, movement
	Max. velocity lower lip up (fear)	−0.61	Facial, movement
	Abs. max. jerk lower lip (emotional pain)	−0.57	Facial, movement
	Max. acc. lower lip down (emotional pain)	−0.53	Facial, movement
	Abs. max. acc. lower lip (emotional pain)	−0.48	Facial, movement
Suicide	PPT (hope and fear)	0.71, 0.55	Speech, timing
	Max. velocity lower lip down (fear)	0.61	Facial, movement
	Avg. lip aperture (SIT)	−0.77	Facial, mouth
	Avg. half mouth surface area right (SIT)	−0.57	Facial, mouth
	Avg. eyebrow displacement (anger)	−0.38	Facial, eyes

The respective interview question or task is shown in parentheses.

Individuals with a GAD-7 score ≥10 reveal a different voice quality compared to controls expressed by a higher harmonics-to-noise ratio when speaking about hope, fear and secrets. Moreover, cases showed reduced movement and facial expression indicated by smaller lip aperture in the SIT task, slower lip movements in terms of velocity, acceleration and jerk measures, in particular while speaking about emotional pain and fear. In line with these findings, we found a statistically significant negative correlation between average absolute acceleration of the lower lip for the fear task and GAD-7 total scores (*p*: 0.007, *r*: –0.320). For the cohort with a suicidal risk ≥*Moderate*, we observed a higher percent pause time while speaking about fear and hope than for controls. In addition, we detected reduced movement and facial expression for cases than controls captured by less eyebrow displacement when asked about anger, lip opening in the SIT task (*p*: 0.004, *r*: –0.332) and lower maximum downwards velocity of the lower lip when speaking about fear.

### 3.2. User feedback

#### 3.2.1. Survey

**Figure 5:**
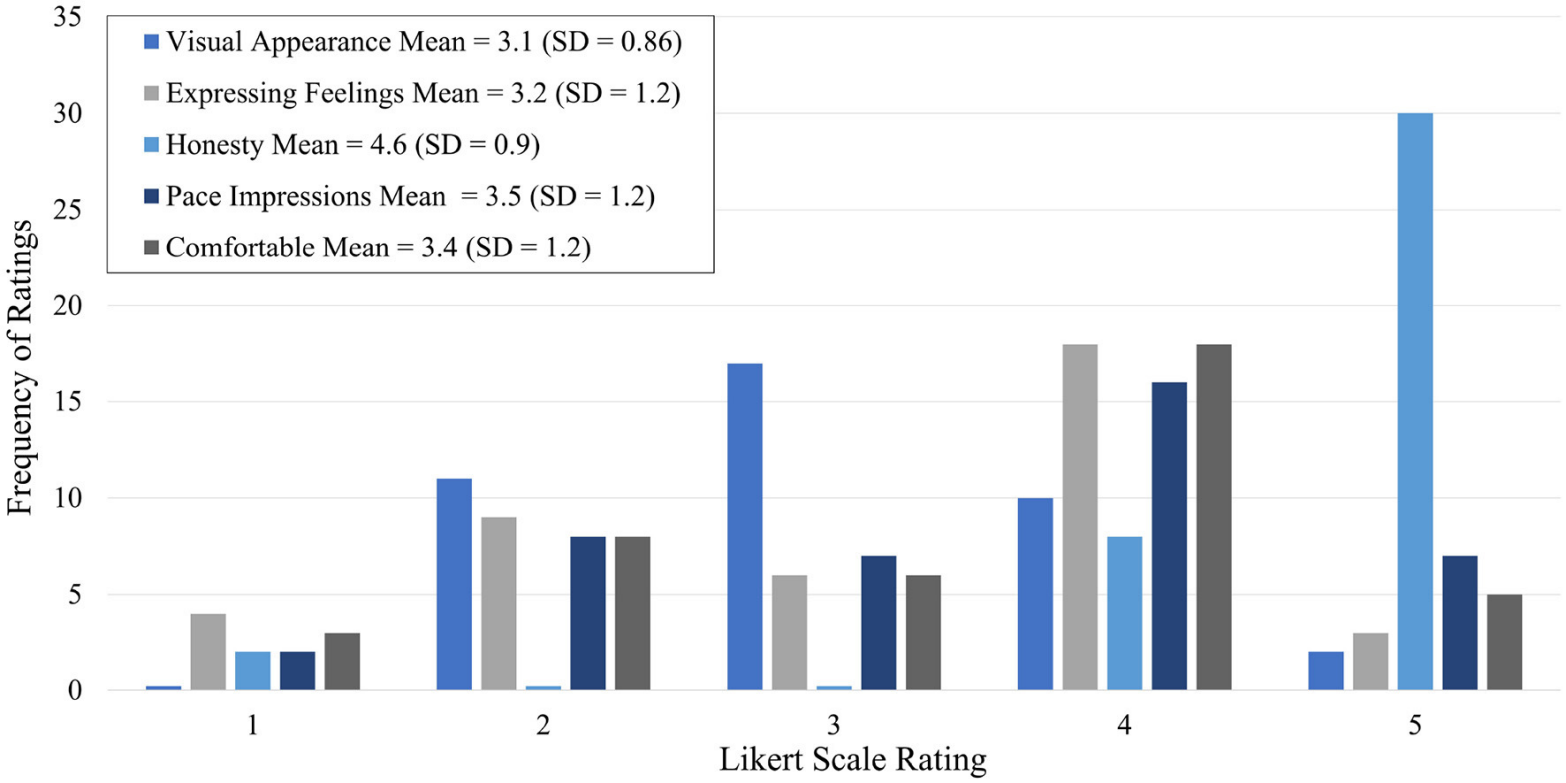
Survey distribution.

Forty participants (59%) completed the five-question Likert scale survey about their experience, shown in Table 3. Frequency distributions, means, and standard deviations of the responses are shown in Figure 5. Student *t*-tests yielded no significant difference between case and control Likert scale ratings for all questions, for all conditions, except between suicide risk cases and controls for question four (*p* = 0.03), which asks about the virtual assistant's appearance and voice. Participants who scored “Moderate” risk or above on the C-SSRS Screener were more likely to rate Tina's visual appearance and voice higher compared to controls.

#### 3.2.2. Likes/advantages

The most frequent theme among the things that participants liked about the dialog agent was a “comfortable experience” (*N* = 43, 60%). One user reported: “I am very impressed by the realism of the experience. It felt almost as if I were talking to a real human being... the voice was pleasant and felt calming.” The second-most recurrent theme was “accessibility” (*N* = 24, 32%). Another respondent stated: “There's value in screening for immediate risk when people aren't available.” The third commonly occurring theme was “confidentiality” (*N* = 17, 22.7%), yet another participant commented: “I felt that I was able to be more open because it wasn't a real person; I didn't feel as though anyone was judging me.”

#### 3.2.3. Dislikes/disadvantages

The most common theme was “lack of human likeness” (*N* = 62, 82.7%). Users felt “awkward with the conversation flow” and were concerned about the virtual agent's “ability to understand nuances in someone's tone.” The second theme was “perception of lack of risk intervention” (*N* = 3, 4%). One participant stated: “If somebody is in crisis, they wouldn't be caught in time to keep them safe.”

#### 3.2.4. Improvements

The most frequent theme was “interview flow” (*N* = 13, 17.3%). Users felt the pressure to speak for a certain time. A respondent conveyed: “I felt like I was grasping at straws trying to make up more things to say.” The second-most occurring theme was “diversity in prompts” (*N* = 12, 16%). A user suggested to have “more specific questions based on responses.” The third common theme was “different visual” (*N* = 9, 12%). One respondent recommended: “have an option on what kind of voice/face to interact with.” Another user suggested: “it would be helpful to have an avatar that moved and blinked. It would feel less hollow.”

## 4. Discussion

In this study, we explored the potential of using an MDS to collect speech, facial, and semantic text information to aid in the detection of depression, anxiety, and suicidal risk. Most participants indicated they honestly shared their feelings with the virtual agent and found the experience comfortable, highlighting the potential acceptability of this approach. However, participants also identified areas for improvement in the conversational agent, such as the need for more contextually appropriate responses, indicating that further refinement of this methodology is needed.

While previous work has examined using MDSs with participants with depression and anxiety (DeVault et al., 2014; Cummins et al., 2015; Neumann et al., 2020), few studies have included individuals with an elevated suicide risk, which pose unique safety concerns. Indeed, some studies avoid any suicide-related questions and use the PHQ-8 (Kroenke et al., 2009) rather than the PHQ-9 (Kroenke et al., 2001), which skips the last question about suicide risk. In this study, participants received resources such as the 988 Suicide and Crisis Lifeline, and CRCs observed the interaction live and were able to follow the safety contingency plan if an acute risk arose. As several participants noted, there are limits to the degree a system such as this could immediately intervene. This is a valid concern and is true of any remote screening or patient monitoring system for suicide risk. Whether used clinically or in future studies where direct observation is not possible, safeguards will need to be in place to address this concern.

During feature analysis, we found many features agreed with previous clinical mental health ML studies. Our findings for depression are in line with other work that found a higher PPT (Cannizzaro et al., 2004; Mundt et al., 2012; Bennabi et al., 2013) or an increase in total pause time associated with lower total speech duration (Albuquerque et al., 2021) for individuals with depression, as well as a correlation between clinical condition and standard deviation of F0 and PPT (Åsa Nilsonne, 1987). The lower standard deviations of the F0 for cases with a PHQ-9 ≥10 compared to controls indicate less variation in speech.

In agreement with our findings, previous studies have reported different voice quality in individuals with anxiety disorder compared with control participants, measured as harmonics-to-noise ratio (Murray and Arnott, 1993; Siegman and Boyle, 1993). The findings, however, show an irregular trend (increase vs. decrease). Moreover, as suggested by our results, a lower HNR score in controls seems to be counter-intuitive, as a low HNR is associated with a higher degree of hoarseness (Yumoto et al., 1982), which refers to abnormal voice quality (Feierabend and Shahram, 2009). Anxiety may be more intensely manifested in facial features, as our study suggests that (a) the facial modality performs better than speech and language features in classifying cases with anxiety disorder vs. controls and (b) twelve facial features are consistently selected across experiments compared to only one speech feature. Our results indicate that adults affected by this disorder show reduced facial behavior. However, anxiety disorders and especially facial features on this are understudied (Low et al., 2019), and more research is needed to investigate multimodal markers of the disease.

As in depression, a higher PPT for individuals with suicidal risk ≥*Moderate* has been observed, which was also shown in clinician-patient interaction (Venek et al., 2015). Regarding the PPT, the largest effect between cases and controls is found when talking about hope, as can be seen in Table 7, suggesting individuals at moderate or high suicidal risk struggle more with this topic. In addition, decreased facial activity, as evidenced by lower eyebrow displacement and mouth opening in our study, has been associated with higher suicide risk in previous research studies (Galatzer-Levy et al., 2020). Cases show a higher downward, but not upward, velocity of the lower lip compared to controls, which may be interpreted as a more abrupt opening of the mouth compared to controls. However, future investigations are needed to provide a more thorough understanding of the observed behaviors.

The text features shown in Table 5 are the top 10 case and control features by weight of linear SVMs fit to the entire dataset for each condition, and represent a fraction of the total number of features. A full linguistic analysis is out of scope here, however, there are some noteworthy observations. First, other studies have found personal pronouns related to depression and suicide risk (Chung and Pennebaker, 2007), yet no personal pronouns appear in Table 5. For depression, the appearance of a name as a case feature is likely related to the limited number of depression cases in this study. For anxiety, the word “anxious” appears as a top feature for cases, while “depression” appears as a control feature. Interestingly, for suicide, some of the control features could be associated with stressors or protective factors related to suicide risk, depending on context. The use of n-grams (contiguous sequence of n nummber of words) or more advanced NLP techniques, such as word embeddings (Mikolov et al., 2013; Pennington et al., 2014), could capture more nuanced aspects of language. In a clinical setting, tools such as Local Interpretable Model-Agnostic Explanations (LIME; Ribeiro et al., 2016) could improve the interpretability of text features by considering their impact per prediction, as opposed to globally, and displaying the features within the context they were used.

For the classification tasks, we found that the combination of modalities typically improved model performance for all tasks. This is not altogether surprising as one might expect more information to help classification performance. However, an exception to this was observed for the depression classification task with facial features, which was not run due to the low number of significant features. It is likely that facial features would improve model performance with a larger, more balanced sample.

It is also worth noting that not all our single-modality models performed above the chance level, with five out of eight delivering near-chance results. We elected to report these lower-performing models as they are are also informative and contribute to a comprehensive understanding of the dataset and the performance characteristics of the different modalities. This approach underscores the importance of having a sufficient number of significant features for reliable classification performance.

Text features performed the best when identifying suicide risk and performed with near chance levels for the identification of depression and anxiety. The interview questions of the MHSAFE interview (hopes, secrets, anger, fear, and emotional pain) were originally developed to screen for suicide risk (Pestian et al., 2010), therefore, classification performance for depression and anxiety may improve if questions more relevant for those conditions are added. The nature of the interaction may also have influenced the semantic content shared by participants which may have affected classifier performance, as some indicated a pressure to speak long enough to fill the required amount of time.

As the prevalence of mental health conditions increases amidst greater health system strain, digital approaches to screen and monitor these conditions are emerging as promising avenues for research. Our interviews, which on average took <10 min, suggest the possibility of providing clinically useful information for three conditions, given that models have been appropriately validated. However, these are early findings and further research is needed to confirm and expand upon our results. The results of the interview could potentially offer a new perspective as clinical decision support for difficult cases, or direct appropriate resources or referrals to individuals when a mental health professional is not available.

### 4.1. Limitations and future directions

Although these findings align with the earlier-discussed studies with regards to the identification of important features and general model discriminative ability, some limitations should be noted. First, studies with small sample sizes face inherent limitations, such as limited representation of different genders and races, which may impact generalizability. Additionally, small sample size ML studies may lead to overly optimistic estimates of classification performance as it is difficult to eliminate information leakage across folds when both train and test sets are used for feature selection (Vabalas et al., 2019; Berisha et al., 2022). Our method to determine the number of features to include during the classification tasks was based on the number of features identified as statistically significant when fit on the entire dataset. Therefore, we acknowledge some information leakage across the folds, however, the specific features selected were determined during each CV fold, and as seen in Table 7, only a fraction of the statistically significant features from the entire dataset (Figure 3) appear in all of the CV folds. While this technique may have lead to more reasonable estimates of model performance, we intend to repeat our analysis with a larger sample size in future work and ultimately explore more advanced modeling techniques, including deep learning. Note that we did not explore deep learning methods in this work, for two important reasons—the primary one being the need to clearly interpret the results/performance of the system in order to be practically applicable in the healthcare setting, and the second being the limited sample size. Lastly, we tried oversampling techniques to account for our dataset's case imbalance, but did not see any improvements; we will continue to explore these techniques with a larger dataset.

The supervision of participants by CRCs during this study may have influenced participant responses. Previous research indicates that participants interacting with a computer reported lower fear of self-disclosure and displayed more intense sadness than when they believed they were interacting with a human (Gratch et al., 2007; Lucas et al., 2014; Rizzo et al., 2016). In our study, some participants even pointed out the potential advantage of system confidentiality, and none expressed negative feedback regarding the presence of CRCs. The identification of features consistent with the literature and the discriminative ability of the classifiers suggest that most participants expressed themselves at least as openly as in studies involving human interviewers. In future studies, we aim to remove direct CRC supervision to better reflect real-world scenarios of remote patient monitoring and to possibly elicit more authentic user responses. By doing so, we also hope to facilitate the collection of larger datasets, crucial for overcoming common machine learning challenges such as overfitting, generalizability, and bias.

Participants indicated several areas of improvement in the user feedback section that we have implemented and will test in future studies. First, we have added slight animation of the virtual agent with the aim of increasing human likeness. To improve the flow of the interview and aid in prompting participants, we have reduced the minimum amount of time required for each response to 30 s and included nudges specific to each question of the MHSAFE interview.

## 5. Conclusions

This study found that a multimodal dialog system (MDS) is a feasible, scalable, and interpretable solution for remote patient monitoring (RPM) in real-world clinical depression, anxiety and suicidal populations. A novelty of this study is that it investigates features derived from multiple modalities—speech, language, and facial behavior—to analyze and characterize three mental disorders—depression, anxiety, and suicide risk—simultaneously. An interesting finding to highlight here is that different modalities were found to be most effective at distinguishing controls from cases for each disorder considered: speech for depression, facial for anxiety, and text/language for suicidality. We also found that a combination of features from different modalities extracted during a brief, standardized MDS interview generally improved the discriminative ability of machine learning models for mental state characterization in *all three disorders*. Furthermore, both healthy participants and those affected by a mental disorder indicated acceptance of the technology. Finally, we presented several lessons learned from implementation, user experience, feature engineering and machine learning perspectives for future practitioners.

## Data availability statement

The datasets presented in this article are not readily available because the dataset contains confidential health-related data that cannot be shared. These data will be made available for research purposes only to any researcher who meet criteria for access to confidential data based on relevant Institutional Review Boards. Requests to access the datasets should be directed to research@clarigenthealth.com.

## Ethics statement

The studies involving humans were approved by Advarra's Institutional Review Board. The studies were conducted in accordance with the local legislation and institutional requirements. The participants provided their written informed consent to participate in this study.

## Author contributions

JC, VRi, MN, and VRa conceptualized the study and wrote the manuscript. VRi, MN, and DB performed feature analysis and model development/validation. AH and JW-B performed analysis of survey responses. JC and JW-B are principal investigators of the study Classification and Assessment of Mental Health Performance Using Semantics—Expanded. All authors contributed to the article and approved the submitted version.

## Footnotes

^1^Global Health Data Exchange (GHDx), https://ghdx.healthdata.org/ [accessed: 2022-12-20].

^2^Centers for Disease Control, https://www.cdc.gov/nchs/fastats/mental-health.htm [accessed: 2023-07-11].

^3^ https://github.com/rpy2/rpy2

^4^We consider each combination of a speech/facial measure and a task (interview question) as one feature. Twenty-eight percent of speech features (26) and 33% of facial features (146) were removed because of missing data. The majority of these were from the initial SIT sentence and the warm up question.

^5^One out of 68 participants did not specify their sex at birth. This participant's session was excluded from the analysis of speech and facial features.
