# Supplementary material for: A multimodal dialog approach to mental state characterization in clinically depressed, anxious, and suicidal populations
Source: Front Psychol. 2023 Sep 11;14:1135469. doi: 10.3389/fpsyg.2023.1135469 (PMC10520716; doi:10.3389/fpsyg.2023.1135469)
Supplement: Supplementary file 1 [file Data_Sheet_1.PDF]

Supplementary Material

Table S1: Participant descriptive statistics including cases and controls.

Variable	Participants	Sessions	Case Sessions			Control Sessions		
			**PHQ-9** ≥ 10	**GAD-7** ≥ 10	**C-SSRS** $\geq$ **Mod.**	**PHQ-9** < 10	**GAD-7** < 10	**C-SSRS** $< Mod.$
Count (%)	68 (100.0%)	73 (100.0%)	15 (20.6%)	21 (28.8%)	26 (35.6%)	58 (79.5%)	52 (71.2%)	47 (64.4%)
Average Age (SD)	38.8 (14.7)	38.7 (14.7)	39.3 (13.3)	34.5 (13.1)	38.8 (15.7)	38.57 (15.1)	40.42 (15.0)	37.99 (14.3)
Average Interview Length (min) (SD)	9.6 (2.2)	9.3 (2.3)	9.7 (2.5)	9.0 (2.4)	9.7 (2.2)	9.5 (2.2)	9.8 (2.1)	9.7 (2.2)
Average Word Count (SD)	917.0 (302.1)	925.0 (309.9)	912.1 (374.2)	899.1 (308.5)	964.1 (316.2)	928.3 (209.9)	935.4 (309.9)	936.1 (304.2)
**Sex**								
Male (%)	15 (22.1%)	16 (21.9%)	3 (4.11%)	6 (8.2%)	9 (12.3%)	13 (17.8%)	10 (13.7%)	7 (9.6%)
Female (%)	52 (76.5%)	56 (76.7%)	12 (16.4%)	15 (20.6%)	16 (21.9%)	44 (60.3%)	41 (56.2%)	40 (54.8%)
Prefer Not to Answer	1 (1.5%)	1 (1.4%)	- (-%)	- (-%)	1 (1.4%)	1 (1.4%)	1 (1.4%)	- (-%)
**Race**								
White or Caucasian (%)	50 (73.5%)	54 (74%)	12 (16.4%)	17 (23.3%)	21 (28.8%)	42 (57.5%)	37 (50.7%)	33 (45.2%)
Black or African American (%)	10 (14.7%)	11 (15.1%)	2 (2.7%)	3 (4.1%)	2 (2.7%)	9 (12.3%)	8 (11.0%)	9 (12.3%)
Asian (%)	5 (7.4%)	5 (6.9%)	- (-%)	1 (1.4%)	- (-%)	5 (6.8%)	4 (5.5%)	5 (6.8%)
Other (%)	3 (4.4%)	3 (4.1%)	1 (1.4%)	- (-%)	3 (4.1%)	2 (2.7%)	3 (4.1%)	- (-%)
**Income (USD)**								
<$25k (%)	16 (23.5%)	18 (24.7%)	7 (9.6%)	9 (12.3%)	5 (6.8%)	11 (15.1%)	9 (12.3%)	13 (17.8%)
$25K-$34.9K (%)	9 (13.2%)	9 (12.3%)	1 (1.4%)	1 (1.4%)	5 (6.8%)	8 (11.0%)	8 (11.0%)	4 (5.5%)
$35K-$49.9K (%)	7 (10.3%)	7 (9.6%)	2 (2.7%)	2 (2.7%)	1 (1.4%)	5 (6.8%)	5 (6.8%)	6 (8.2%)
$50K-$74.9K (%)	6 (8.8%)	6 (8.2%)	1 (1.4%)	1 (1.4%)	4 (5.5%)	5 (6.8%)	5 (6.8%)	2 (2.7%)
$75K-$99.9K (%)	8 (11.8%)	8 (11.0%)	1 (1.4%)	1 (1.4%)	2 (2.7%)	7 (9.6%)	7 (9.6%)	6 (8.2%)
$100K-$149.9K (%)	8 (11.8%)	10 (13.7%)	1 (1.4%)	3 (4.1%)	4 (5.5%)	9 (12.3%)	7 (9.6%)	6 (9.6%)
>$150K (%)	10 (14.7%)	11 (15.1%)	2 (2.7%)	4 (5.5%)	4 (5.5%)	9 (12.3%)	7 (9.6%)	7 (9.6%)
Prefer Not to Answer (%)	3 (4.4%)	3 (4.1%)	- (-%)	- (-%)	- (-%)	3 (4.1%)	3 (4.1%)	3 (4.1%)
Missing (%)	1 (1.5%)	1 (1.4%)	- (-%)	- (-%)	1 (1.4%)	1 (1.4%)	1 (1.4%)	- (-%)
**Education**								
High School or Equivalent (%)	2 (2.9%)	2 (2.7%)	- (-%)	- (-%)	2 (2.7%)	2 (2.7%)	2 (2.7%)	- (-%)

Continued on next page

Table S1 – Continued from previous page

			Case Sessions			Control Sessions		
**Variable**	**Participants**	**Sessions**	**PHQ-9** ≥ 10	**GAD-7** ≥ 10	**C-SSRS** $\geq$ **Mod.**	**PHQ-9** < 10	**GAD-7** < 10	**C-SSRS** $< Mod.$
Some College, no degree (%)	14 (2.6%)	14 (19.2%)	5 (6.8%)	7 (9.6%)	3 (4.1%)	9 (12.3%)	7 (9.6%)	11 (15.1%)
Associate Degree (%)	4 (5.9%)	6 (8.2%)	3 (4.1%)	4 (5.5%)	4 (5.5%)	3 (4.1%)	2 (2.7%)	2 (2.7%)
Bachelors (%)	27 (39.7%)	29 (39.7%)	6 (8.2%)	5 (6.8%)	10 (13.7%)	23 (31.5%)	24 (32.9%)	19 (26.0%)
Masters (%)	15 (22.1%)	16 (21.9%)	1 (1.4%)	4 (5.5%)	5 (6.8%)	15 (20.5%)	12 (16.4%)	11 (15.1%)
Doctorate (%)	6 (8.8%)	6 (8.2%)	- (-%)	1 (1.4%)	2 (2.7%)	6 (8.2%)	4 (6.8%)	4 (5.5%)

End of table

Table S2. Description of speech features.

Feature	Description
**Signal-to-noise ratio**	strength of the desired signal relative to background noise (undesired signal); proxy for speaking volume
**Percent pause time**	proportion of the total duration of all pauses to the total duration of the utterance
**Speaking duration**	total duration of an utterance
**Articulation duration**	total duration of an utterance without pauses
**Harmonics-to-noise ratio**	ratio between periodic components and non-periodic components comprising a segment of voiced speech; captures how clean or noisy a speech signal is
**Fundamental frequency**	number of times a sound wave produced by the vocal cords repeats during a given time period; perceived as the pitch of a voice
**Shimmer**	amplitude variation of the voice produced by the vocal cords from cycle to cycle
**Jitter**	pitch variation of the voice produced by the vocal cords from cycle to cycle

Table S3. Description of facial features. LL: lower lip, JC: jaw center.

Feature	Description
**Lip aperture**	Euclidean distance between the upper lip and lower lip (center)
**Lip width**	Euclidean distance between right and left corners of the mouth
**Mouth surface area**	the total mouth surface area, calculated as the sum of the area of the two triangles with vertices RC (right corner of the mouth), UL (upper lip center), LL (lower lip center) and LC (left corner of the mouth), UL, LL
**Mouth symmetry ratio**	mean symmetry ratio of the left and right sides of the mouth, measured as the ratio of the left to the right surface area of the mouth
**Velocity of LL/JC**	velocity of LL/JC is calculated as the first derivative of LL/JC path with time
**Acceleration of LL/JC**	acceleration of LL/JC is calculated as the second derivative of LL/JC path with time
**Jerk of LL/JC**	jerk of LL/JC is calculated as the third derivative of LL/JC path with time
**Eye blinks/sec.**	The number of eye blinks in blinks per second is calculated using the eye aspect ratio
**Eye opening**	eye opening is calculated as the Euclidean distance between upper and lower extremities of each eye
**Eyebrow displacement**	vertical eyebrow displacement is calculated as the vertical distance between the center of the eyebrow and the center of the inter-caruncular distance for each eyebrow
